# Primary Ewing's Sarcoma of the Spine in a Two-Year-Old Boy

**DOI:** 10.1155/2016/8027137

**Published:** 2016-11-08

**Authors:** Ali J. Electricwala, Jaffer T. Electricwala

**Affiliations:** Electricwala Hospital and Clinics, Himalayan Heights, Pune, Maharashtra 411013, India

## Abstract

Ewing's Sarcoma (ES) is a highly malignant bone tumour. It may involve any part of the skeleton but the most frequent parts are the ilium and diaphysis of femur and tibia (Alfeeli et al., 2005; Zhu et al., 2012). Primary ES of the spine is extremely rare (Yan et al., 2011). It accounts for only 3.5 to 14.9 percent of all primary bone sarcomas. The age of presentation ranges from 12 to 24 years (median 21 years) (Ferguson, 1999; Sharafuddin et al., 1992; Klimo Jr. et al., 2009). We report an unusual case of primary ES of the spine in a two-year-old boy, who presented to us with paraparesis and features of cauda equina syndrome. MRI scan showed a tumour mass arising from the pedicle of L4 vertebra invading the spinal canal. Tc-99 bone scan showed increased tracer uptake in L4 vertebra and normal tracer uptake elsewhere in the skeleton. After reaching the diagnosis of a space occupying lesion invading the lumber spinal canal, we performed a decompressive laminectomy and a biopsy was sent which confirmed the diagnosis of ES. Immunohistochemistry showed tumour cells staining positive for CD-99 (specific stain for ES). Gene testing showed an EWS-FLI 1 chimera. Surgery was followed by good improvement in motor signs. The child was then referred to a specialized oncotherapy centre for further treatment, radiation, and chemotherapy. To the best of our knowledge, we are the first to report primary ES of the spine at the age of two years.

## 1. Introduction 

Ewing's Sarcoma (ES) is a highly malignant bone tumour. It may involve any part of the skeleton but the most frequent parts are the ilium and diaphysis of femur and tibia [1, 2]. Primary ES of the spine is extremely rare [3]. It accounts for only 3.5 to 14.9 percent of all primary bone sarcomas. The age of presentation ranges from 12 to 24 years (median 21 years) [4–6].

## 2. Case History

A two-year-old male child, first issue of a nonconsanguineous marriage, was brought with a 15-day history of progressive weakness of both the lower extremities, difficulty in standing and walking, and progressive loss of bowel and bladder function. He had no history of trauma, back pain, and failure to thrive. No constitutional symptoms were present. He had no significant past, personal, or family history. All developmental milestones were achieved for his age.

On clinical examination there was paraspinal fullness and complete loss of power below the level of the knee joint in both lower extremities. (Hip flexion and knee extension was grade 5; ankle dorsiflexion, great toe extension, and ankle planter flexion were grade zero.) Bulk was normal and tone was reduced. There were decreased sensations below the level of L3 dermatome in both the lower limbs. Perianal sensations were reduced. Ankle reflex and planter (Babinskis) response were absent bilaterally.

His laboratory parameters were normal except for a raised erythrocyte sedimentation rate (48 mm/hour). X-rays of the whole spine, chest, and abdomen were normal. Ultrasonography of the abdomen and pelvis showed abnormal distension of the bladder suggestive of the possibility of neurogenic bladder.

**Figure 1:**
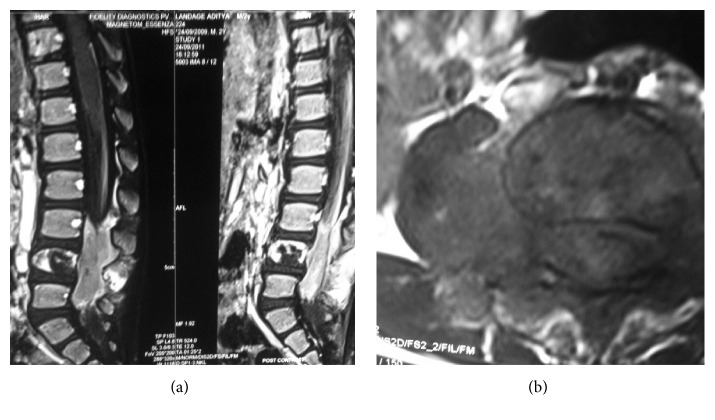
Sagittal projection of postcontrast T1 and T2 images demonstrating destruction of L4 vertebral body and tumour mass invading the spinal canal and posterior elements at L3, L4, and L5 vertebral levels. Axial projection of postcontrast T2 MRI image demonstrating tumour mass arising from the pedicle of L4 vertebra invading the spinal canal and the Right Psoas muscle.

MRI of the lumbosacral spine (Figures 1(a) and 1(b)) revealed a soft tissue mass arising from the pedicle of L4 vertebral body invading the spinal canal, posterior elements, and Right Psoas muscle with destruction of the L4 vertebral body.

**Figure 2:**
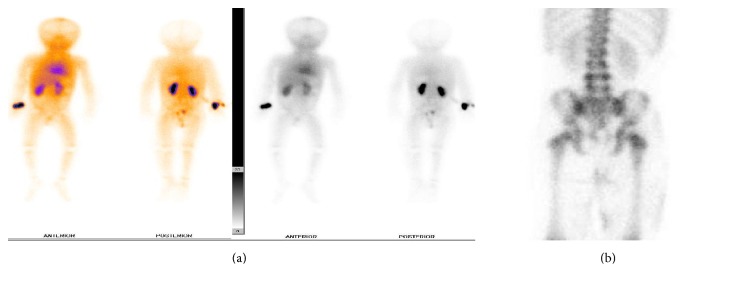
Tc-99 labelled bone scan.

The patient was without the evidence of metastasis at presentation as found by chest and abdominal radiographs, chest computed tomography scan, and Tc-99 bone scan (Figures 2(a) and 2(b)).

**Figure 3:**
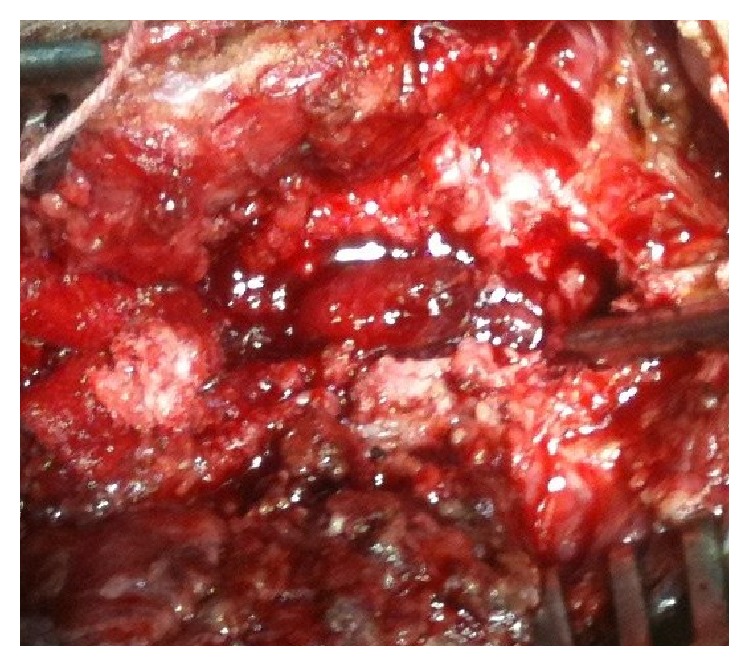
Decompressive laminectomy done revealing the tumour mass invading the spinal canal.

The child underwent a decompressive laminectomy (Figure 3) as the first line of management.

**Figure 4:**
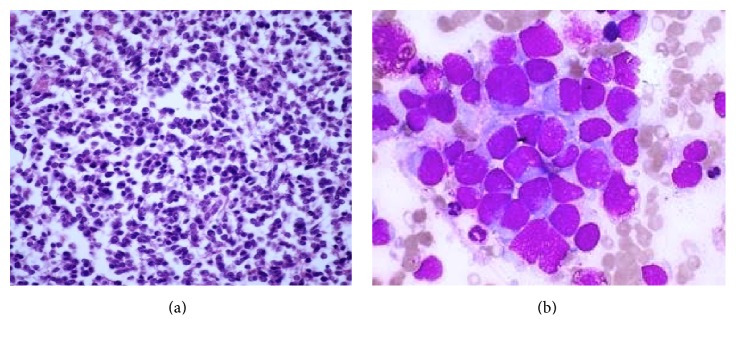
Low power view showing small round cells uniformly packed in nests. High power view showing tumour cells.

**Figure 5:**
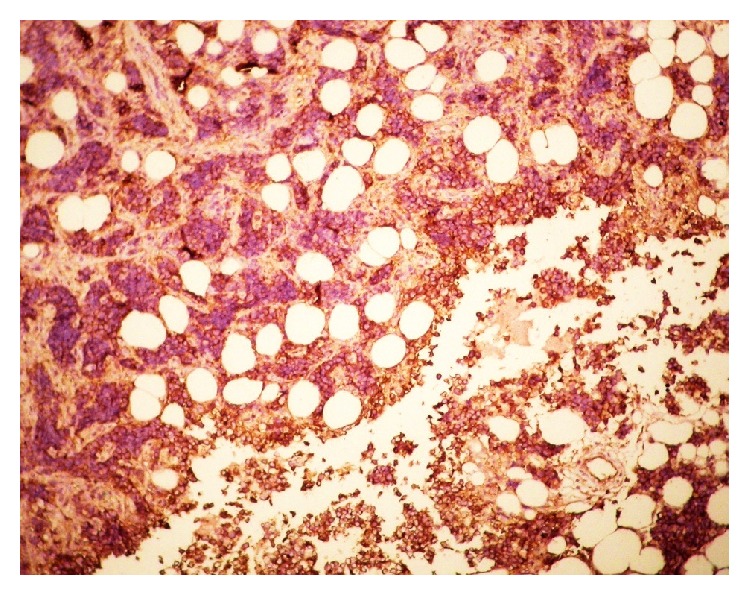
Immunohistochemistry stain showing tumour cells staining positive for CD-99.

The diagnosis of Ewing's Sarcoma was confirmed on histopathology, immunohistochemistry, and cytogenetic analysis. Histopathology showed small round cells packed in nests (Figures 4(a) and 4(b)). Immunohistochemistry showed tumour cells staining positive for CD-99: specific stain for ES (Figure 5). Gene testing showed an EWS-FLI 1 chimera.

Following decompressive surgery the patient had a good initial improvement in motor weakness. On postoperative day 15 the patient was referred to a specialized oncotherapy centre for radiation and combination chemotherapy.

## 3. Discussion

Ewing's Sarcoma (ES) is a small round cell tumour and accounts for one quarter of all primary bone tumours during childhood. Its peak incidence is during the second decade of life and it is very rare after 30 years of life [7]. ES usually presents with pain and swelling of the affected bone and vertebral involvement occurs in less than 5 percent of cases [8]. It has a poor prognosis but multimodality chemotherapy has increased life expectancy by 40 percent. Primary ES of the spine is a very rare condition [9]. Our case report is an extremely rare case of primary ES of the spine in a two-year-old boy. Our case was diagnosed 3 days after presentation. The initial interpretation of the MRI scan by the radiologist was that of a destructive lesion in the vertebral body of the fourth lumber vertebra most likely to be due to an infective process like tuberculosis.

In a retrospective study of Widhe et al., at the first visit, a bone tumour was suspected in only 19 percent of the cases of primary ES of the spine [10, 11]. A high index of suspicion and careful physical examination is required for the diagnosis of this condition. Signs of spinal cord compression may be the only initial indicators for primary ES of the spine [11–13].

Histopathology is the mainstay of diagnosis of small round cell tumours. The differential diagnoses of small round cell tumours include neuroblastoma, primitive neuroectodermal tumours of bone (PNET), malignant lymphoma, rhabdomyosarcoma, and ES. The differentiation between these tumours on the basis of light microscopy alone is not accurate.

Current standards require evaluation by immunohistochemistry (CD-99) and cytogenetic analysis for the diagnosis of ES [14–17]. Chromosomal data from ES reveals a remarkably consistent chromosomal anomaly: the reciprocal translocation t(11;22)(q24;q12) involving chromosome 22 located on EWS-FLI 1 in more than or equal to 90% of the cases [18, 19]. The child in this case report satisfied both the histological and cytogenetic criteria required for the diagnosis.

Radiographs usually show a lytic lesion but sometimes sclerotic changes are also seen. However these findings on X-ray appear late usually after neurological signs have become obvious [15].

MRI scan is more sensitive than CT in the early detection on ES [20, 21]. Bone scan before staging is an important step to rule out other foci and in the follow-up treatment of primary ES of the spine [22].

These tumours have variable sensitivity to radiation and chemotherapy due to biological heterogenecity [23]. The classical chemotherapy regimen followed in ES consists of VAC-A (vincristine sulfate, dactinomycin, cyclophosphamide, and doxorubicin hydrochloride) [24, 25].

## 4. Conclusion

The purpose of this study was to report the incidence of such a rare tumour in a very young child. To the best of our knowledge, we are the first to report primary ES of the spine at the age of two years. Orthopaedic surgeons may encounter such a condition and should have a high index of suspicion to diagnose this rare tumour at its early stage for a better prognosis.
